# French validation of brace questionnaire

**DOI:** 10.1186/1748-7161-9-S1-O51

**Published:** 2014-12-04

**Authors:** Julie Deceuninck, Jean-Claude Bernard

**Affiliations:** 1 CMCR des Massues, Lyon, France

## Background

Quality of Life (QoL) Scales have to be introduce in the treatment evaluation of our patients with adolescent idiopathic scoliosis.

Vasiliadis and al. create the Brace Questionnaire (BrQ), the one which is specific for brace treated adolescents. This tool was developed and validated in Greek.

## Material and methods

The BrQ is made of 34 items on Likert Scale, divided in 8 domains. The questionnaire was developed in order that the child could fill in it alone and is adapted for 9 to 18 years old. The lowest scale is 20 and the best 100. The highest scales show a better QoL.

The process of cultural adaptation of the questionnaire was in accordance with the International Quality of Life Assessment (IQOLA) Guidelines.

## Statistical analysis

Firstly descriptive statistics will be used to calculate mean scores and standard deviations for a given question and a domain. The second level will be comparative concerning reliability and validity.

